# "Hands of fame": a new tool to improve dedication of key opinion leader for hand hygiene

**DOI:** 10.1186/1753-6561-5-S6-P107

**Published:** 2011-06-29

**Authors:** AF Widmer, H Schuhmacher, S Tschudin-Sutter, the infection control team

**Affiliations:** 1 Infectious disease and Hospital epidemiology, University of Basel, Basel, Switzerland

## Introduction / objectives

Compliance of hand hygiene is frequently less than optimal and heavily depends on the dedication of the key opinion leaders. There are few tools to support their role model for hand hygiene. Dedicated days, information campaigns and similar activities lead to tremendous, but short-lived impact on the hand hygiene compliance. Psychological studies have shown, that a public statement improves compliance with the statement. We therefore searched for a tool who to trigger a public long-term statement of the key opinion leaders of the hospital.

## Methods

The University of Basel hospitals is a 900 bed tertiary care center with 5 Intensive CUs and kidney and bone marrow transplant program. In January 2011, all key opinion leaders of the university hospital were asked to participate in an action to imprint both their hands on a flagstone of 80x80cm. The composition of the flagstone is a composite plastic, that allows to imprint the hands, and is fixed within one hour. In addition, the names are placed on the flagstone to indicate.

## Results

Of the 21 key opinion leaders, 20 participate in the study. Flagstone with the hands of the individuals were placed in the main entrance of the hospital restaurant. The names of each participant is placed on the flagstone in copper letters to remind the individual as the healthcare workers of proper hand hygiene. The half-life of the 40kg flagstone (total of 880kg transported) is approximately, 25 years.

## Conclusion

Imprinted flagstones placed in the hospital area allows to motivate key opinion leaders for hand hygiene. The “hands of fame” flagstones are a new tool to remind each opinion leader on a daily basis on their commitment for hand hygiene. The long-half life of flagstones will likely exceed the lifetime of the individual.

## Disclosure of interest

None declared.

